# The neutrophil-to-lymphocyte ratio is associated with mortality in the general population: The Rotterdam Study

**DOI:** 10.1007/s10654-018-0472-y

**Published:** 2018-12-19

**Authors:** Jesse Fest, T. Rikje Ruiter, Bas Groot Koerkamp, Dimitris Rizopoulos, M. Arfan Ikram, Casper H. J. van Eijck, Bruno H. Stricker

**Affiliations:** 1 Department of Surgery, Erasmus MC University Medical Center, Rotterdam, The Netherlands; 2 Department of Epidemiology, Erasmus MC University Medical Center, PO Box 2040, 3000 CA Rotterdam, The Netherlands; 3 Department of Biostatistics, Erasmus MC University Medical Center, Rotterdam, The Netherlands

**Keywords:** Low-grade inflammation, Neutrophil-to-lymphocyte ratio, Mortality

## Abstract

Inflammation is a risk factor for morbidity and mortality in the elderly. The neutrophil-to-lymphocyte ratio (NLR) is a marker of systemic inflammation that integrates the information of the leukocyte differentials into one variable. We aimed to assess whether the NLR is a risk indicator for overall and cause-specific mortality in the general population. We analyzed data (2002–2014) from the Rotterdam Study, a long-standing, population-based, prospective cohort study in a community-dwelling ageing population. The association between the NLR and time to all-cause mortality was assessed with Cox proportional hazard models. We additionally assessed cardiovascular, cancer and other mortality. The multivariable analyses were adjusted for age, gender, socio-economic status (SES), smoking status, body mass index, type 2 diabetes, and history of cancer and cardiovascular disease (CVD). Data of 8715 individuals were included. The mean age was 65.9 years (SD 10.5) and the majority were women (57.1%). The NLR was higher in men, higher age categories, smokers and among individuals with lower SES, prevalent diabetes, or a history of cancer or CVD. During the 11.7 years follow-up period, 1641 individuals died. Survival among individuals in the 3rd, 4th, and 5th quintile of the NLR was significantly poorer than that of those in the 1st quintile (*P* < 0.001). In the multivariable analysis, NLR levels were independently and significantly associated with an increased risk of all-cause mortality (HR 1.64; 95% CI 1.44–1.86), cardiovascular mortality (HR 1.92; 95% CI 1.49–2.48), and other mortality (HR 1.86; 95% CI 1.54–2.24). No significant association was found for cancer mortality (HR 1.20; 95% CI 0.95–1.51). The NLR is a strong and independent risk indicator for mortality in the elderly population. Its clinical value needs to be established in further studies.

**Electronic supplementary material:**

The online version of this article (10.1007/s10654-018-0472-y) contains supplementary material, which is available to authorized users.

## Introduction

Inflammation is considered an important risk factor for morbidity and mortality in the elderly. It is still largely unclear whether we may speak of a causal relation between inflammation and mortality, or whether the inflammation is a manifestation of an underlying illness that causes early death. Moreover, the inflammatory markers are known to increase with age, therefore an elevation of these markers may also be ‘part of the process of ageing’ [1].

C-reactive protein (CRP) has been extensively studied as a marker of inflammation and more specifically as a risk indicator for cardiovascular, cancer, and all-cause mortality [2–5]. Nevertheless, no conclusive evidence has been found on its potential causal role in mortality of any cause and its clinical use for early identification of patients at risk of cardiovascular disease [2, 4, 6]. Furthermore, CRP is likely to be just one of many different elements in the inflammatory pathway.

In an attempt to gain more insight into the relationship between inflammation and mortality, also the total leukocyte count has been studied. It has previously been shown that it is related to cardiovascular, cancer, as well as all-cause mortality [7–10]. However, the total leukocyte count encompasses several cell types, such as granulocytes, lymphocytes and monocytes, which potentially all play a different role [11]. Granulocytes, as a whole, or more specifically neutrophils, are associated with a negative influence on survival, whereas lymphocytes are considered to have protective effects on survival [11–13]. While analyzing them together would not appreciate the opposite roles they seem to have, analyzing them apart would not account for the interaction between these subtypes in their association with mortality.

The neutrophil-to-lymphocyte ratio (NLR) is a composite marker of absolute peripheral neutrophil and lymphocyte counts, which can be used to study the effects of both simultaneously [14]. It is a well-studied marker for survival in patients with cancer and in patients with cardiovascular disease [13, 14]. However, it is unknown whether it also is predictive of cancer, cardiovascular, or all-cause mortality in the general population. To this end we studied the NLR and its potential association with overall and cause-specific mortality within the context of the Rotterdam Study; a long-standing, population-based, prospective cohort study among a community-dwelling ageing population, with detailed information on illness and risk factors for chronic disease. We hypothesized that an increased NLR is independently associated with mortality in apparently healthy individuals.

## Methods

### Study design and population

The rationale and design of the Rotterdam Study have previously been described [15, 16]. Briefly, from 1989 to 1993, inhabitants of the suburb of Ommoord in the city of Rotterdam, aged 55 years and older, were invited to participate. Of 10,275 invited subjects, 7983 participated (78%). A second cohort of 3011 persons, also aged 55 years and older, (response: 67%) was enrolled in the years 2000 and 2001. In 2006, the study was again extended with 3932 persons aged 45 years and older (response: 65%). This resulted in an overall study population of 14,926 individuals aged 45 years and above.

Baseline NLR values were calculated at the earliest study center visit at which a leukocyte differential count was available: the fourth visit of the first cohort (2002–2004; n = 3550), the second visit of the second cohort (2004–2005; n = 2468) and the first visit of the third cohort (2006–2008; n = 3932).

Individuals who had not proved consent for blood draw (N = 1038) were excluded as well as individuals with missing granulocyte, lymphocyte or platelet counts (N = 197).

The Rotterdam Study has been approved by the institutional review board (Medical Ethics Committee) of the Erasmus Medical Center and by the review board of The Netherlands Ministry of Health, Welfare and Sports.

### Assessment of the neutrophil-to-lymphocyte ratio

Fasting blood samples were collected at the study center and full blood count measurements were performed immediately after blood draw. These measurements included absolute counts of granulocytes and lymphocytes and were performed using the COULTER^®^ Ac·T diff2™ Hematology Analyzer (Beckman Coulter, San Diego, California, USA).

The neutrophil-to-lymphocyte ratio (NLR) was calculated on the basis of absolute peripheral granulocyte (as a proxy for the absolute neutrophil count) (N; × 10^9^/Liter) and lymphocyte (L; x10^9^/Liter) blood counts, using the formula: NLR = N/L [14].

The NLR was non-normally distributed and therefore log-transformed prior to performing any of the analyses.

### Assessment of other covariates

Data on the following known independent prognostic factors of mortality were collected at baseline: age, gender, socio-economic status (SES; based on education level [high/intermediate/low]), baseline body mass index (BMI; kg/m^2^), smoking status [never/former/current], prevalent type 2 diabetes status (DM; based on a fasting plasma glucose level of ≥ 7.0 mmol/L (≥ 126 mg/dL) or non-fasting plasma glucose level of ≥ 11 mmol/L (≥ 200 mg/dL) or use of blood glucose medication), history of cancer (based on pathology), and lastly, history of cardiovascular disease, including transient ischemic attacks (TIA), stroke (CVA), myocardial infarction (MI), and coronary revascularization (percutaneous transluminal coronary angioplasty or coronary artery bypass grafting) [17–19].

High-sensitivity CRP measurements (mg/ml; using a particle enhanced immunoturbidimetric assay, Roche Diagnostics, Mannheim, Germany) were available in a subgroup of the study.

### Assessment of outcome

The main outcome of this study was time to all-cause mortality. Dates of death were obtained through the mortality registry of the municipality and the causes of death were obtained from general practitioners’ records or hospital discharge letters. The causes of death were coded independently by two physicians according to the ICD- 10 and the ICPC-2 [20, 21].

### Statistical analysis

For each participant, follow-up started at the day of inclusion and ended at the date of death or end of the study period (1st of January 2014), whichever came first.

Participants were divided into five groups based on the level of the NLR calculated at baseline. Differences between the five groups were assessed with ANOVAs for normally distributed continuous variables and *χ*^2^-tests for categorical variables. Kaplan–Meier plots were calculated for quintiles and extreme quantiles of the NLR and compared with Log-Rank tests.

Proportional hazard models were used to assess the association between the NLR levels at baseline (continuously and in quartiles) and time to all-cause mortality. Subsequently we assessed the association for cardiovascular and cancer mortality, respectively.

For most variables the proportional hazard assumption did not hold. Therefore, follow-up time was divided into five strata (< 2 years, 2–4 years, 4–6 years, 6–8 years and > 8 years). For example: an individual with an event after 5.4 years follow-up, contributed follow-up time to the first (2 years), second (2 years) and third stratum (1.4 years). The risk of mortality in the last stratum is therefore conditional upon the survival up until that time [22]. We also performed a traditional proportional hazard regression, the results of which can be interpreted as the averaged risks over time [22].

For 5421 individuals we had a second measurement available, which we included in a multiple measurements analysis using a time-varying covariates in a Cox model [23].

All potential confounders, mentioned above, were assessed individually and were included in the multivariable model when they changed the point estimate by more than 10% or were considered as clinically relevant [24]. The results are reported as hazard ratios (HR) and 95% confidence intervals (CI). Effect modification was assessed for smoking by adding an interaction variable to the model and was considered statistically significant at a *P* value < 0.10. We tried to quantify the presence of any unknown and therefore unmeasured confounding through calculating the E-Value [25].

All statistical analyses were performed using SPSS software (Version 21.0) and R (Version 3.1.3); significance was accepted for two-sided *P*-values at < 0.05.

## Results

### Population characteristics

Data of 8715 participants were included in the analyses (see Supplementary Fig. 1). The mean age was 65.9 years; the majority were women (4980; 57.1%, see Table 1). During an average follow-up period of 7.7 years (maximum follow-up period was 11.7 years), a total of 1641 (18.2%) participants died, of whom 496 from the consequences of cancer (30.2%) and 401 from cardiovascular disease (24.4%). The remaining 45.4% died from another cause such as: chronic obstructive pulmonary disease (COPD), a pneumonia, as a consequence of an accidental fall or multi-comorbidity including Parkinson’s Disease and Alzheimer’s Disease.

**Table 1:** Baseline characteristics for each quintile of the NLR

Characteristic		Total	Neutrophil-to-lymphocyte ratio					*P*-value
			Q1	Q2	Q3	Q4	Q5	
			< 1.30	1.30–1.59	1.60–1.91	1.92–2.41	> 2.41	
	Total	8715	1799	1747	1685	1745	1739	
		N (%)	N (%)	N (%)	N (%)	N (%)	N (%)	
Gender	Male	3735 (42.9)	595 (33.1)	687 (39.3)	703 (41.7)	837 (48.0)	913 (52.5)	< 0.001
	Female	4980 (57.1)	1204 (66.9)	1060 (60.7)	982 (58.3)	908 (52.0)	826 (47.5)	
Age (years)	Mean (SD)	65.9 (10.5)	63.2 (9.7)	64.2 (9.5)	65.4 (10.5)	66.8 (10.6)	70.1 (10.6)	< 0.001
Smoking*	Current	1734 (19.9)	300 (16.7)	341 (19.5)	365 (21.7)	375 (21.5)	353 (20.3)	< 0.001
	Former	4291 (49.2)	854 (47.5)	860 (49.2)	792 (47.0)	876 (50.2)	909 (52.3)	
	Never	2571 (29.5)	627 (34.9)	527 (30.2)	513 (30.4)	458 (26.2)	446 (25.6)	
SES*	High	1652 (19.0)	369 (20.5)	333 (19.1)	321 (19.1)	332 (19.0)	297 (17.1)	0.001
	Intermediate	3598 (41.3)	789 (43.9)	718 (41.1)	690 (40.9)	717 (41.1)	684 (39.3)	
	Low	3348 (38.9)	612 (34.0)	683 (39.1)	654 (38.8)	666 (38.2)	733 (42.2)	
BMI* (kg/m^2^)	Mean (SD)	27.1 (4.2)	27.0 (3.9)	27.1 (4.1)	27.3 (4.1)	27.1 (4.2)	26.9 (4.3)	0.081
DM status	Yes	952 (10.9)	145 (8.1)	169 (9.7)	179 (10.6)	221 (12.7)	238 (13.7)	< 0.001
	No	7763 (89.1)	1654 (91.9)	1578 (90.3)	1506 (89.4)	1524 (87.3)	1501 (86.3)	
History of cancer	Yes	688 (7.9)	131 (7.3)	131 (7.5)	124 (7.4)	121 (6.9)	181 (10.4)	0.001
	No	8027 (90.9)	1668 (92.7)	1616 (92.5)	1561 (92.6)	1624 (93.1)	1558 (89.6)	
History of CVD	Yes	789 (9.1)	106 (5.9)	121 (6.9)	143 (8.5)	159 (9.1)	260 (15.0)	< 0.001
	No	7926 (90.1)	1693 (94.1)	1626 (93.1)	1542 (91.5)	1586 (90.9)	1479 (85.0)	

Differences between the five groups were assessed with ANOVAs for normally distributed continuous variables and *χ*^2^-tests for categorical variables

*SES* socio-economic status, *DM* diabetes mellitus type 2, *CVD* cardiovascular disease

*Unknown: Smoking (119; 1.4%), SES (117; 1.3%) and BMI (167; 1.9%)

Baseline characteristics for the total population and for each quintile of the NLR can be found in Table 1. In summary, the male gender, a higher age, a lower SES, smoking habit, prevalent diabetes, prior cancer diagnosis, and a history of cardiovascular disease were all associated with a higher NLR.

### Main outcome

**Fig. 1:**
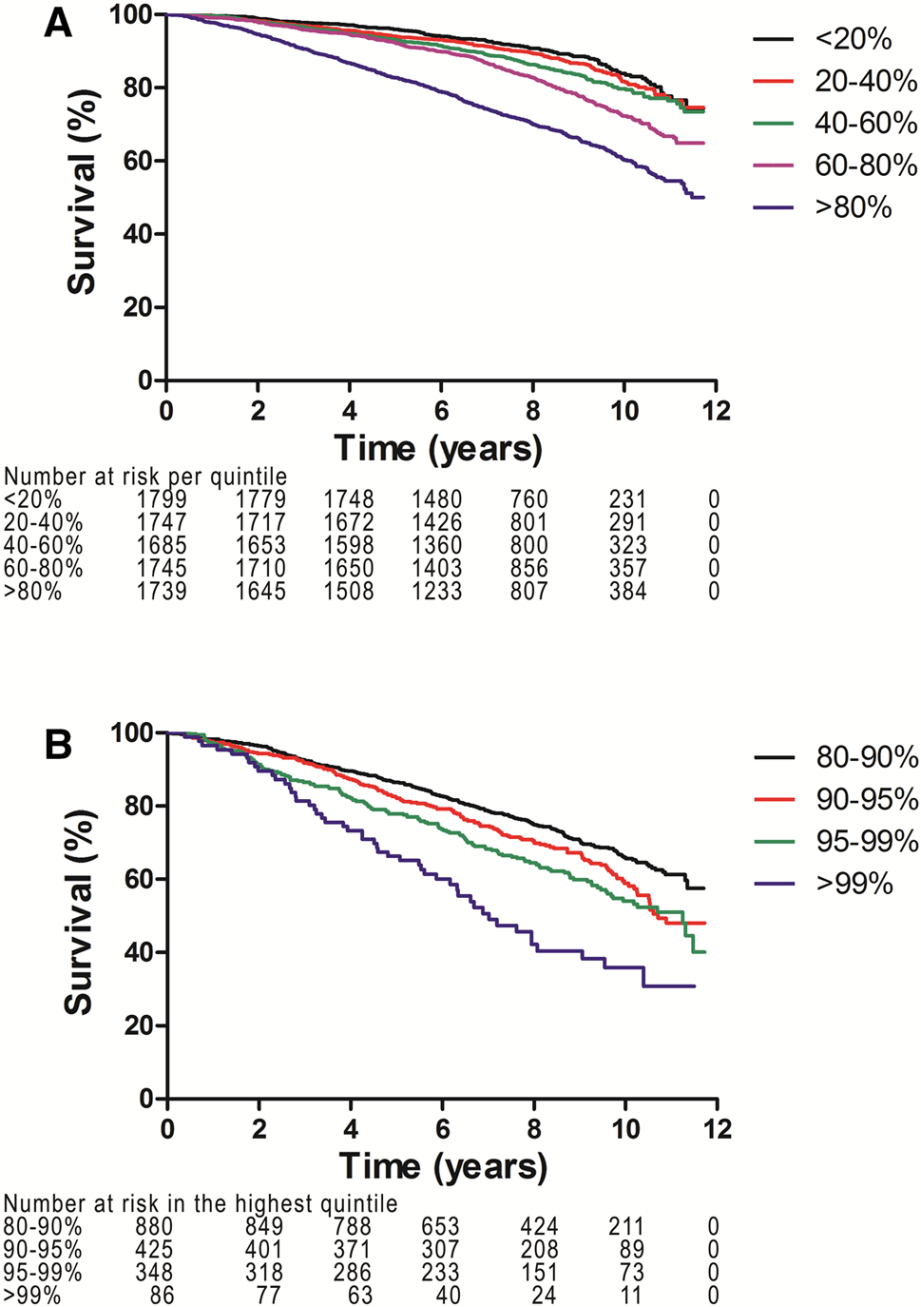
**a** Kaplan–Meier curves for all-cause mortality for each quintile of the NLR (*P*-value < 0.001). **b** Kaplan–Meier curves for all-cause mortality for the highest quintile of the NLR (*P*-value < 0.001)

The overall survival was poorer for participants in the higher quintiles of the NLR than for those in the lowest one (Logrank test: *P*-value < 0.001, see Fig. 1a). Survival of participants in the 2nd quintile was not significantly different from that of participants in the 1st quintile (reference), but for other quintiles it did differ significantly. In a further analysis which was restricted to the highest quintile, survival for the 1% with the highest NLR levels was worst (Logrank test: *P*-value < 0.001, see Fig. 1b).

Multivariable analysis showed that the NLR was independently associated with all-cause mortality, after adjusting for age, gender, SES, BMI, smoking, DM, and history of CVD and cancer. The effect of the NLR was not modified by smoking. On average the risk was increased by 64% (HR 1.64; 95% CI 1.44–1.86). The E-values for this analysis were 2.17 for the point estimate and 1.89 for the confidence intervals, respectively. The observed HR of 1.64 could be reduced to 1.00 if there was an unmeasured confounder with a risk of 2.17 or above.

In the stratified analysis, the risk was higher in each subsequent quartile, with a significantly higher risk in the fourth quartile in comparison to the lowest quartile (HR 1.59, 95% CI 1.37–1.86), with a significant trend over the quartiles (*P*-value < 0.001, see Table 2).

**Table 2:** Cox proportional hazard regression for the association of the NLR and all-cause mortality

Events/cohort	NLR	HR	Lower 95% CI	Upper 95% CI
1551/8352	Logtransformed	1.64	1.44	1.86
226/2107	Q1	Reference	–	–
274/2073	Q2	1.05	0.88	1.25
374/2082	Q3	1.13	0.96	1.33
677/2090	Q4	1.59	1.37	1.86

Adjusted for: gender, age in years, *SES* (socio-economics status: high/intermediate/low), smoking status (current/former/never), *BMI* (body mass index: kg/m^2^), *DM* (type 2 diabetes mellitus status), history of cancer and history of cardiovascular disease. *P*-value for trend over quartiles < 0.001

In a sensitivity analysis in which we allowed the NLR the change over time for individuals with a second measurement, the averaged risk for all-cause mortality was increased with 68% (HR 1.68; 95% CI 1.48–1.90) in the fully adjusted model.

**Fig. 2:**
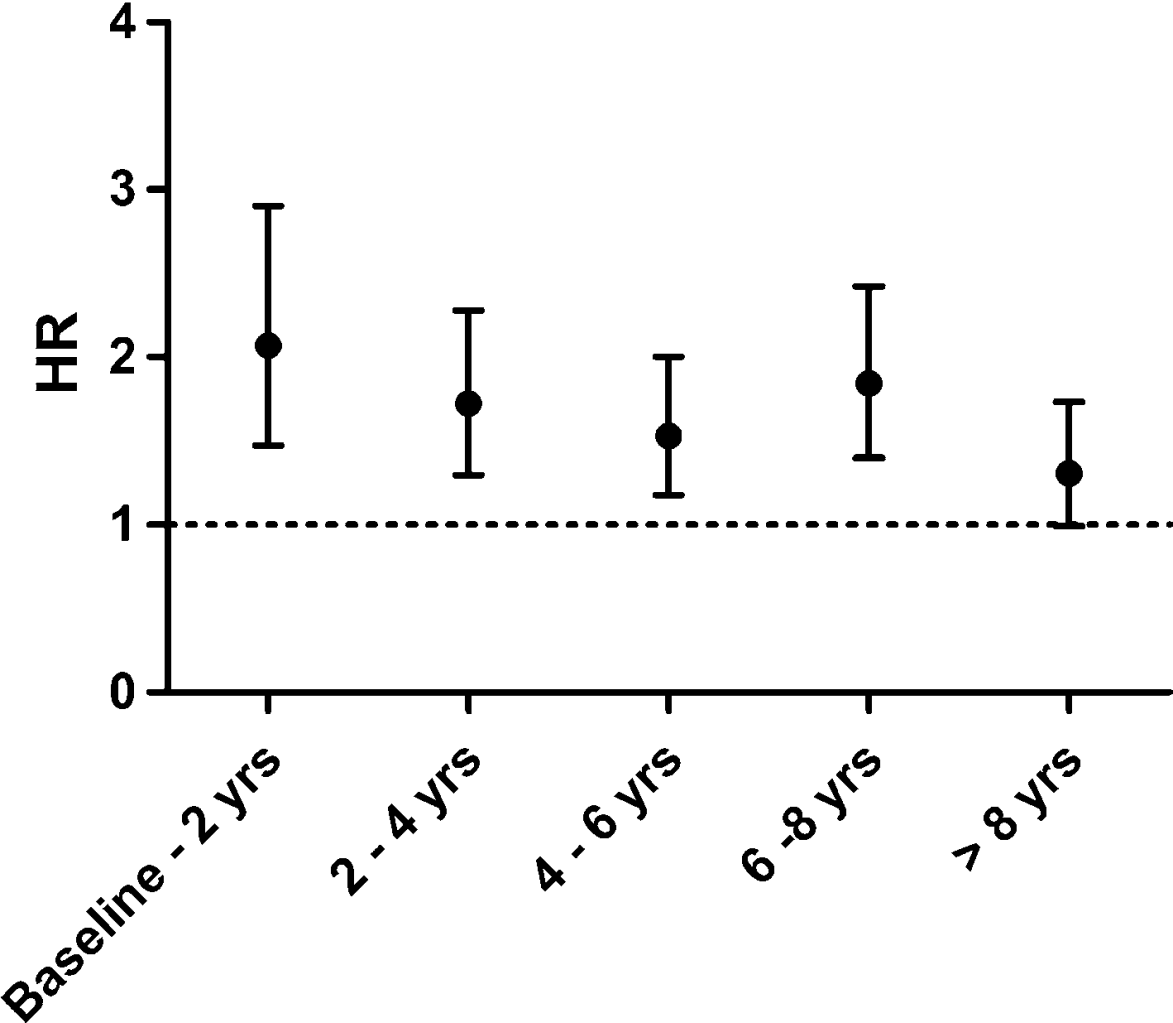
Risk of NLR-related all-cause mortality over time. Adjusted for: sub-cohort, gender, age (in years), socio-economic status (high/intermediate/low), smoking status (current/former/never), BMI (body mass index, kg/m^2^), prevalent type 2 diabetes mellitus, history of cardiovascular disease and history of cancer. Risk for each time stratum were for: baseline–2 years (HR 2.07, 95% CI 1.47–2.90), 2–4 years (HR 1.72, 95% CI 1.30–2.28), 4–6 years (HR 1.53, 95% CI 1.18–2.00), 6–8 years (HR 1.84, 95% CI 1.40–2.42) and > 8 years (HR 1.31, 95% CI 0.99–1.73)

The hazard ratio was highest within the first two years after baseline, in which individuals with a higher NLR level at baseline had a more than twofold risk to die of any cause (HR 2.07, 95% CI 1.47–2.90). The hazard ratio gradually decreased over time, but the NLR remained. associated with an increased risk, albeit non-significantly, of 31% for those with a follow-up time of > 8 years (HR 1.31, 95% CI 0.99–1.73) (see Fig. 2).

Subsequently, we assessed whether the association between baseline inflammatory markers and mortality was attenuated by CRP. A CRP measurement was available for 3457 individuals from RS-III. CRP levels were independently associated with all-cause mortality, but the association was no longer significant when the NLR was also added to the multivariable model. The point estimate of the NLR was not attenuated by adding CRP to the model (see Table 3).

**Table 3:** Cox proportional hazard regression for the association of the NLR and all-cause mortality, additionally adjusted for CRP, in a sub-population of the cohort

Clinical variable	Main model + NLR			Main model + CRP			Main model + NLR + CRP		
	HR	Lower 95% CI	Upper 95% CI	HR	Lower 95% CI	Upper 95% CI	HR	Lower 95% CI	Upper 95% CI
Female	0.75	0.52	1.08	0.72	0.50	1.04	0.78	0.54	1.13
Age (in years)	1.09	1.07	1.11	1.10	1.08	1.12	1.09	1.07	1.11
SES									
High	Reference			Reference			Reference		
Intermediate	1.27	0.76	2.12	1.21	0.73	2.03	1.21	0.72	2.02
Low	1.74	1.03	2.93	1.57	0.93	2.66	1.56	0.92	2.64
Smoking									
Never	Reference			Reference			Reference		
Former	2.04	1.20	3.49	1.96	1.14	3.36	1.94	1.29	3.32
Current	3.38	1.93	5.93	3.47	1.97	6.11	3.35	1.90	5.91
History cancer	2.56	1.57	4.17	2.60	1.59	4.25	2.59	1.58	4.23
DM	1.37	0.83	2.26	1.44	0.87	2.39	1.44	0.87	2.38
BMI (in kg/m^2^)	0.98	0.94	1.02	0.98	0.94	1.02	0.98	0.94	1.02
NLR	2.04	1.31	3.20	–	–	–	1.92	1.19	3.10
CRP (in mg/ml)	–	–	–	1.20	1.02	1.42	1.12	0.94	1.33

For 3457 individuals from RS-III we had a CRP measurement available, we added CRP to the model to see whether the association between the NLR and the all-cause mortality was attenuated. In RS-III in total 129 individuals died. Proportional hazard assumptions were tested separately in this sub-population and upheld for all variables. History of CVD was neither a significant predictor nor a confounder in this subpopulation and was therefore not included in the model

*HR* hazard ratio, *SES* socio-economic status, *BMI* body mass index, *NLR* neutrophil-to-lymphocyte ratio, *CRP* C-reactive protein, *CVD* cardiovascular disease

### Sub-analyses

Additionally, we addressed cause-specific mortality, assessing possible associations between the NLR at baseline and risk of cardiovascular-, cancer- and, other mortality. The risk for cardiovascular mortality was significantly increased and relatively constant over time, with an average HR of 1.92 (95% CI 1.49–2.48) (Supplementary Fig. 2a). In contrast, no significantly increased risk was observed for cancer related mortality, with an average HR of 1.20 (95% CI 0.95–1.51) (Supplementary Fig. 2B). For other mortality the average risk was significantly increased by 86% (HR 1.86; 95% CI 1.54–2.24). It was highest in the first 2 years with a HR of 4.28 (95% CI 2.44–7.51) and decreased over time to a 31% higher, albeit statistically non-significant, risk for individuals with a follow-up time > 8 years (HR 1.31, 95% CI 0.90–1.91) (Supplementary Fig. 2C).

## Discussion

Previous studies have shown that the NLR is a prognostic marker for mortality in patients with cardiovascular disease and cancer [13, 14]. We hypothesized that the NLR was independently associated with mortality in apparently healthy individuals. To our knowledge, this is the first study confirming this hypothesis of an independent relationship between the NLR and early mortality in the general population.

Multiple studies have investigated the association between the WBC count or the leukocyte differentials and all-cause mortality, but none studied individual cell types in relationship to each other. The NLR integrates the information obtained from the leukocyte differentials and provides the opportunity to simultaneously study the association between neutrophils and all-cause mortality and that between lymphocytes and mortality.

Our results are largely in agreement with the results previously found for the association between the WBC count and overall mortality. The WBC count has been consistently associated with both total and cardiovascular mortality. In our study, the association of the NLR with cancer mortality was much weaker and non-significant. Although this might seem counterintuitive because of the prognostic role of the NLR in people with cancer, it is not unexpected as cancer mortality largely depends on available therapeutic options and cancer type. Again this is consistent with literature on the WBC count and cancer mortality in the general population [26, 27].

The effects are controlled for important confounders such as smoking and a higher BMI or comorbidities, such as a history of cardiovascular disease, diabetes or a history of cancer. It is known that the leukocyte and neutrophil counts are also higher in smokers [7, 28]. We indeed found that smoking is an important confounder. The association remains robust, however, after adjustment for this factor, which implies that only part of the association between the NLR and mortality is explained by smoking. Moreover, there was no effect modification of smoking, meaning that the magnitude of the association was not different in smokers compared to non-smokers. Furthermore, the NLR proved the strongest risk indicator when both CRP and the NLR were included in the model. This means that the association was independent from the relationship between CRP and mortality, which suggests that a potential inflammatory pathway that is explained by CRP, is different from the pathway than the one represented by the NLR.

We tried to quantify the presence of any unknown and therefore unmeasured confounding through calculating the E-Value [25]. Although any residual confounding cannot be completely ruled out, we found that any unknown confounder would have to have a risk of 2.17 or above to explain the observed effect. Considering the large number of confounders we have adjusted for, we believe it is unlikely that the effects in this study can be explained by such strong residual confounding.

Overall, our findings seem to confirm that there is an independent relationship between inflammation and mortality. What the nature of this association is, remains uncertain. Although the relationship might be etiological, it may also be that the NLR is a proxy measure of the ageing process or rather a manifestation of an underlying disease.

Consistent with this latter hypothesis, we found that the NLR-related risk of mortality was highest for the first two years of follow-up and decreased over time. This is explained by the effects seen for other mortality (see Supplementary Fig. 2C) and may be a result of a depletion of individuals with an underlying illness or poor health status. However we controlled for history of cancer and cardiovascular disease and even when the first 8 years of follow-up are excluded, the association still persists, making underlying disease a less likely explanation.

Another explanation may be that of a causal association. For instance, it is known that neutrophils infiltrate atherosclerotic plaques and may play a role in the rupture, resulting in a cardiovascular incident [29.] However, this would mean there is an intermediate between the NLR and mortality and that neutrophils play no role in the actual process of dying.

The last explanation would be that the immune system gets damaged as part of the ageing process and that the NLR is a proxy marker for this biological phenomenon.

### Strengths and limitations

A large population-based and prospective cohort study such as the Rotterdam Study, with a long follow-up period and detailed information on prevalent disease and important risk factors, is the design of choice for studying associations between blood levels of inflammatory markers and all-cause mortality.

The NLR is derived from the leukocyte differentials, which is a stable, well-standardized and inexpensive measurement that reflects systemic inflammation. Still, cut-off values to stratify patients into currently unidentified risk groups are still lacking. These cut-off values are necessary to evaluate the clinical utility of the NLR.

Another limitation of this study is the fact that the total granulocyte count served as a proxy for the total neutrophil count. We assume, however, that this has had little impact on the results as neutrophils are by far the most abundant type of granulocytes [30]. Any resulting misclassification could have led to an overestimation, but it has been conclusively shown that the associations for granulocytes and neutrophils have the same direction and the same effect size [12]. We believe the obtained effect measures are a fair representation of the true effect.

In conclusion, we have demonstrated that the NLR is independently associated with all-cause mortality in the elderly population, after adjustment for traditional risk factors. Its potential value in clinical practice needs to be established in further studies.

## Electronic supplementary material

Below is the link to the electronic supplementary material.

Supplementary material 1 (DOCX 1900 kb)
