# Supplementary material for: The neutrophil-to-lymphocyte ratio is associated with mortality in the general population: The Rotterdam Study
Source: Eur J Epidemiol. 2018 Dec 19;34(5):463–70. doi: 10.1007/s10654-018-0472-y (PMC6456469; doi:10.1007/s10654-018-0472-y)

**Supplementary Figure 1. Flowchart of the study population.**

**
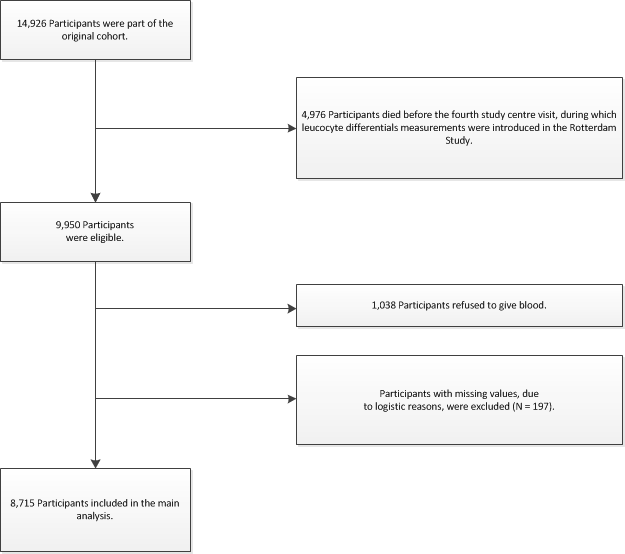
**

**Supplementary Figure 2 A. Risk of NLR-related cardiovascular mortality**


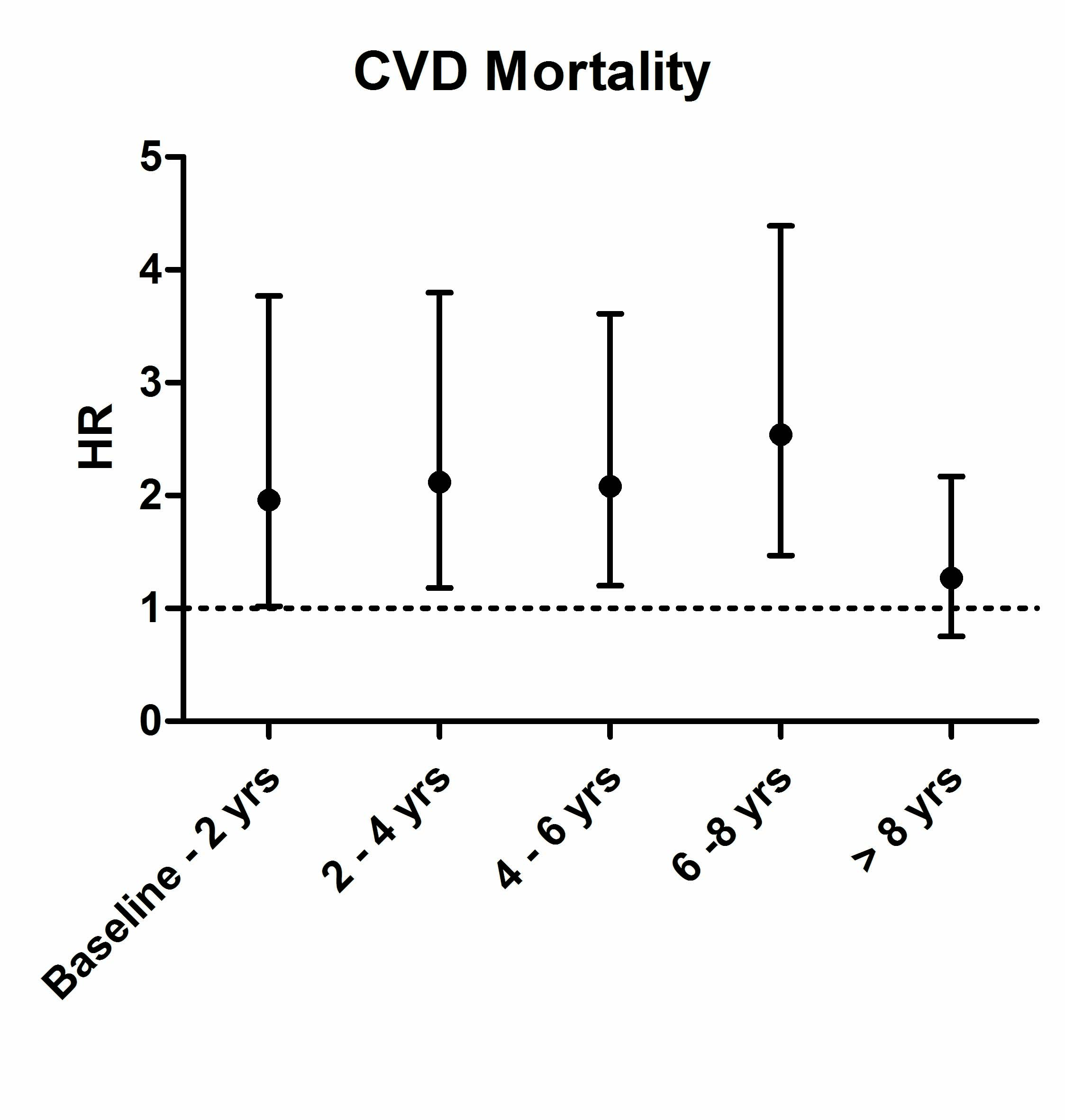


Adjusted for: sub-cohort, sex, age (in years), socio-economic status (high/intermediate/low), smoking status (current/former/never), BMI (body mass index, kg/m^2^), prevalent type 2 diabetes mellitus and history of cardiovascular disease. Risk for each time stratum were for: baseline – 2 years (HR 1.96, 95% CI: 1.02 – 3.77), 2 – 4 years (HR 2.12, 95% CI: 1.18 – 3.80), 4 – 6 years (HR 2.08, 95% CI: 1.20 – 3.61), 6 – 8 years (HR 2.54, 95% CI: 1.47 – 4.39) and > 8 years (HR 1.27, 95% CI: 0.75 – 2.17).

**Supplementary Figure 2 B. Risk of NLR-related cancer mortality**


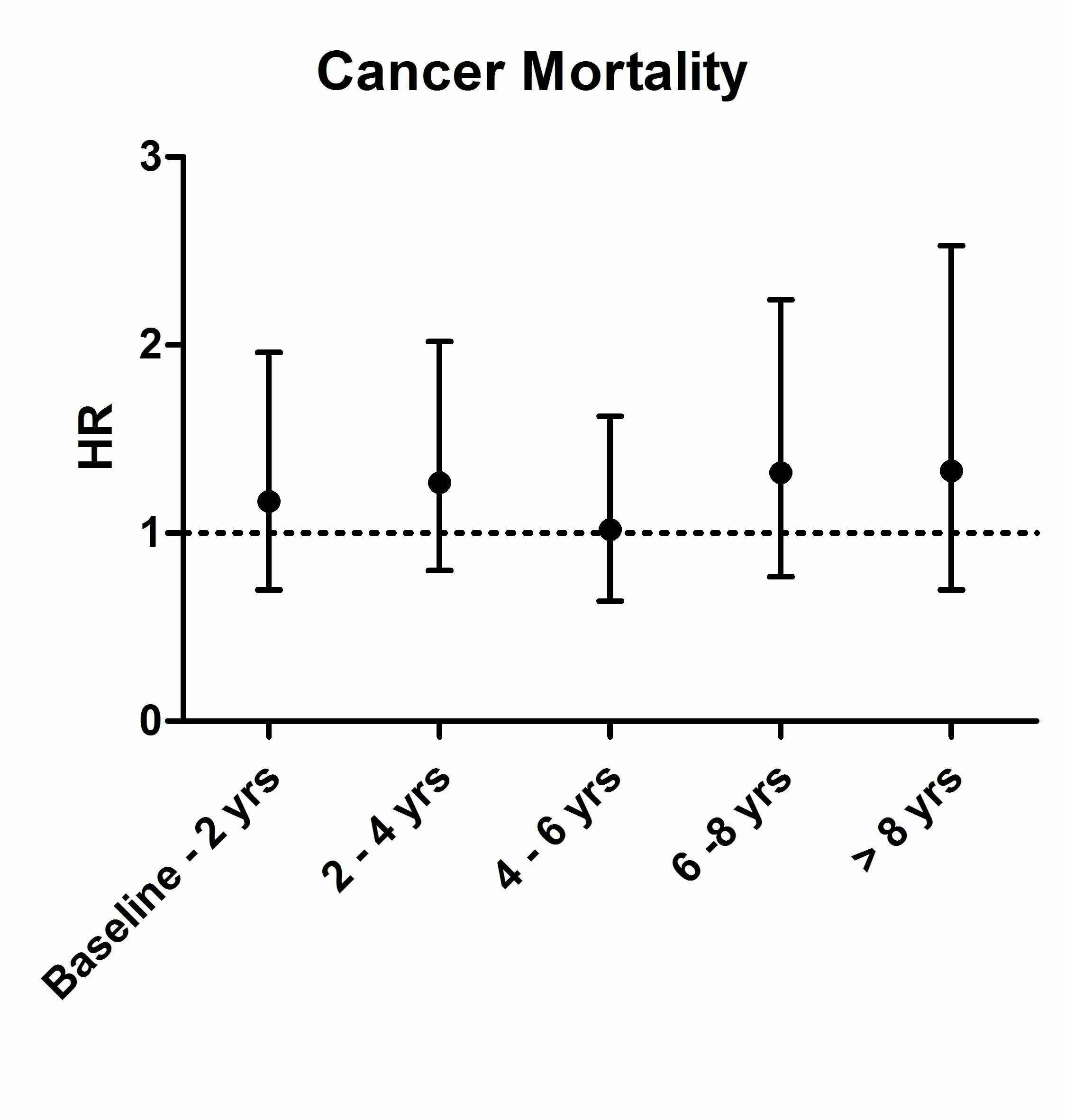


Adjusted for: sub-cohort, sex, age (in years), socio-economic status (high/intermediate/low), smoking status (current/former/never), BMI (body mass index, kg/m^2^), prevalent type 2 diabetes mellitus and history of cancer. Risk for each time stratum were for: baseline – 2 years (HR 1.17, 95% CI: 0.70 – 1.96), 2 – 4 years (HR 1.27, 95% CI: 0.80 – 2.02), 4 – 6 years (HR 1.02, 95% CI: 0.64 – 1.62), 6 – 8 years (HR 1.32, 95% CI: 0.77 – 2.24) and > 8 years (HR 1.33, 95% CI: 0.70 – 2.53).

**Supplementary Figure 2 C. Risk of NLR-related other mortality**


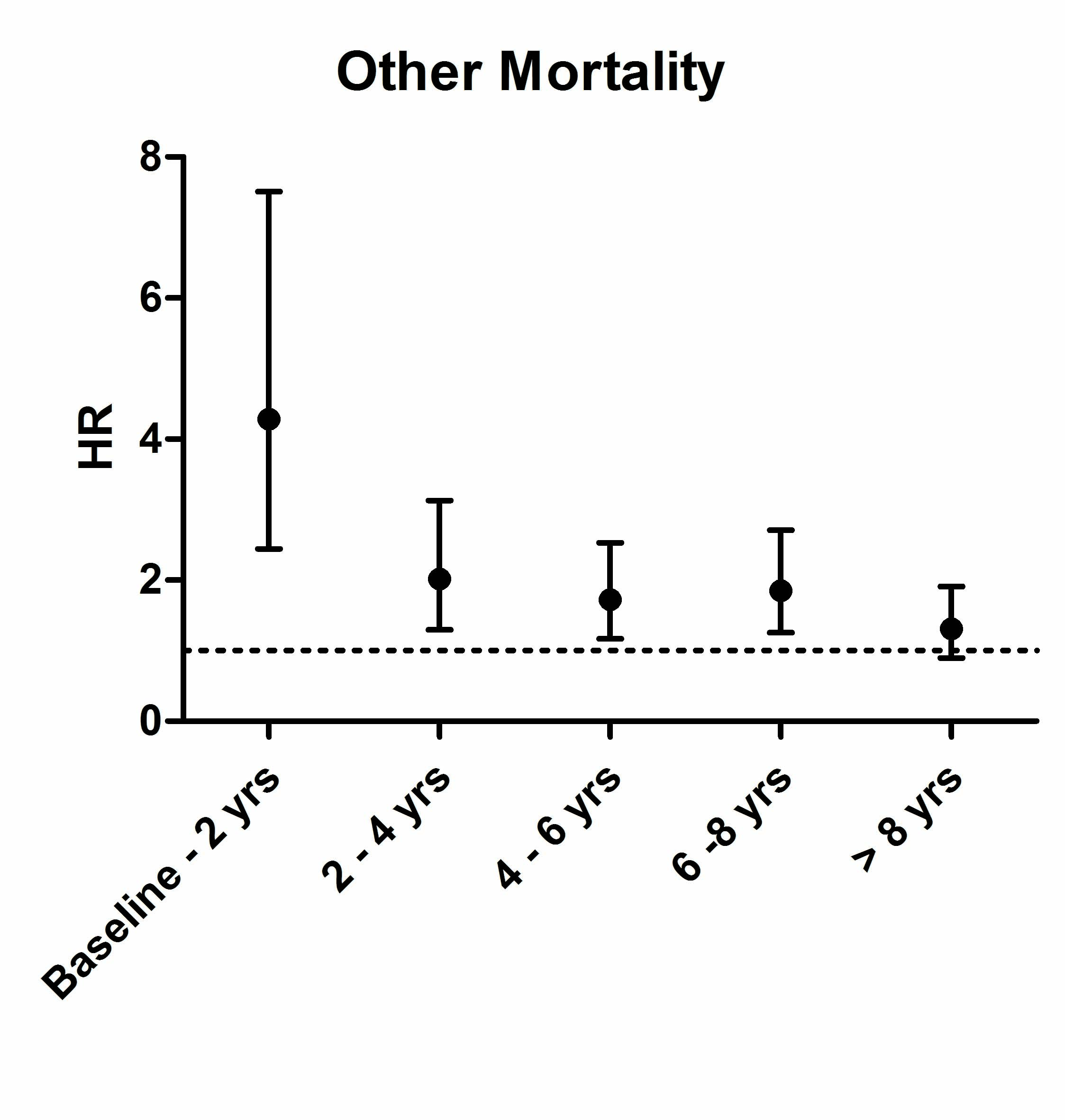


Adjusted for: sub-cohort, sex, age (in years), socio-economic status (high/intermediate/low), smoking status (current/former/never), BMI (body mass index, kg/m^2^), prevalent type 2 diabetes mellitus, history of cardiovascular disease and history of cancer. Risk for each time stratum were for: baseline – 2 years (HR 4.28, 95% CI: 2.44 – 7.51), 2 – 4 years (HR 2.02, 95% CI: 1.30 – 3.13), 4 – 6 years (HR 1.72, 95% CI: 1.17 – 2.53), 6 – 8 years (HR 1.85, 95% CI: 1.26 – 2.71) and > 8 years (HR 1.31, 95% CI: 0.90 – 1.91).

**Supplementary Figure 3. E-value for the average hazard ratio for all-cause mortality.**


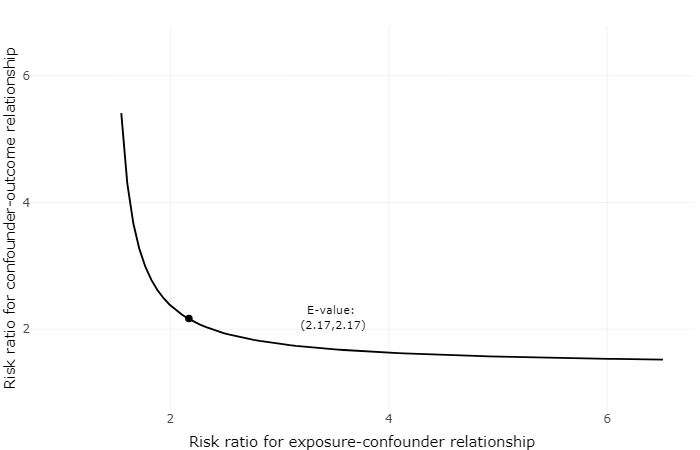

Supplement: Supplementary file 1 — Supplementary material 1 (DOCX 1900 kb) [file 10654_2018_472_MOESM1_ESM.docx]
